# Fast and Accurate Identification of *Candida auris* by High Resolution Mass Spectrometry

**DOI:** 10.3390/jof9020267

**Published:** 2023-02-16

**Authors:** Azadeh Jamalian, Joanna Freeke, Anuradha Chowdhary, G. Sybren de Hoog, J. Benjamin Stielow, Jacques F. Meis

**Affiliations:** 1 Centre of Expertise in Mycology, Radboud UMC/Canisius Wilhelmina Hospital, 6532 SZ Nijmegen, The Netherlands; 2 Medical Mycology Unit, Department of Microbiology, Vallabhbhai Patel Chest Institute, University of Delhi, Delhi 110007, India; 3 Department of Medical Microbiology and Infectious Diseases, Canisius Wilhelmina Hospital, 6532 SZ Nijmegen, The Netherlands; 4 Bioprocess Engineering and Biotechnology Graduate Program, Federal University of Paraná, Curitiba 80060, Brazil; 5 Department I of Internal Medicine, Faculty of Medicine, University of Cologne and Excellence Center for Medical Mycology, University Hospital Cologne, 50931 Cologne, Germany

**Keywords:** *Candida auris*, accurate identification, proteomics clustering, Orbitrap mass spectrometry, medical microbiology diagnostics

## Abstract

The emerging pathogen *Candida auris* has been associated with nosocomial outbreaks on six continents. Genetic analysis indicates simultaneous and independent emergence of separate clades of the species in different geographical locations. Both invasive infection and colonization have been observed, warranting attention due to variable antifungal resistance profiles and hospital transmission. MALDI-TOF based identification methods have become routine in hospitals and research institutes. However, identification of the newly emerging lineages of *C. auris* yet remains a diagnostic challenge. In this study an innovative liquid chromatography (LC)—high resolution Orbitrap^TM^ mass spectrometry method was used for identification of *C. auris* from axenic microbial cultures. A set of 102 strains from all five clades and different body locations were investigated. The results revealed correct identification of all *C. auris* strains within the sample cohort, with an identification accuracy of 99.6% from plate culture, in a time-efficient manner. Furthermore, application of the applied mass spectrometry technology provided the species identification down to clade level, thus potentially providing the possibility for epidemiological surveillance to track pathogen spread. Identification beyond species level is required specially to differentiate between nosocomial transmission and repeated introduction to a hospital.

## 1. Introduction

*Candida auris* was reported for the first time more than a decade ago as a new species causing bloodstream infections in East Asia [1]. This finding was rapidly followed by outbreak reports from India [2], the Middle East, South Africa and South America and soon this pathogen was found to spread globally [3,4,5,6], becoming one of the most common nosocomial fungal pathogens especially in long stay ICU patients [7]. Indeed, *C. auris* has been classified among the 10 most feared fungi [8] and is listed on the CDC urgent threat pathogen list [9]. Although in the early years only sporadic cases of invasive infections due to *C. auris* were reported, outbreaks have occurred on all continents during the last 5 years [10,11,12,13,14,15]. An important factor allowing these outbreaks to occur was inappropriate recognition and misidentification of yeast pathogens in hospital laboratories [16,17]. Accurate and rapid laboratory identification is a basic requirement for much needed source control to mitigate the spread of this multidrug-resistant pathogen in health facilities [18,19]. Whole-genome sequence analyses of a collection of worldwide *C. auris* isolates has shown that at least four and possibly five clades have emerged independently and simultaneously during the last 20 years [20,21]. *Candida auris* appeared to be highly clonal, with five distinct, geographically restricted clades in South Asia (clade I), East Asia (clade II), Africa (clade III), South America (clade IV) and Iran (clade V), that differ from each other by ten-thousands of single-nucleotide polymorphisms [21,22]. At present, *C. auris* isolates belonging to these clades have spread worldwide and have been introduced independently in several countries [23,24,25,26,27]. It is of prime importance for clinical practice to respond quickly with distinction of the clades in view of tracking the source of introduction. This is particularly significant given the differential resistance characteristics of the clades. For example, isolates from the South Asian (clade I) are more than 90% resistant to fluconazole [28] while those from clade II are susceptible. *Candida auris* has several structurally unique mannoproteins [29,30] and sphingolipids [31], characteristics which make them more suited than other *Candida* species to persist on medical devices and in the hospital environment, to form biofilms, to withstand elimination by commonly used disinfectants, and to evade immune responses. Once introduced into a hospital, geographical location or country, *C. auris* can spread rapidly in health care settings. Identification of *C. auris* with MALDI-TOF technology is a well-established and accurate technique at the species level, but in contrast to molecular methods it is unable to differentiate among the five clades [32,33,34,35,36]. In the present paper we used a novel liquid chromatography (LC) Orbitrap^TM^ high resolution mass spectrometry approach for a collection of geographically diverse clinical *C. auris* isolates representing all known geographic clades, and all major genotypes (lineages) within the clades. We describe a novel proof-of-concept methodology for highly accurate *C. auris* identification in the microbiology laboratory, that enables recognition of individual protein mass values (*m/z* values) from each analyzed strain, returning superior identification results with equivalency to those rendered by genomic typing including single nucleotide polymorphism calling methods (e.g., ‘STR’ short-tandem-repeat typing or ‘NGS’ next generation sequencing).

## 2. Materials and Methods

### 2.1. Strain Selection and Sample Material

A total of 102 clinical *C. auris* strains was selected for the analysis to cover all five emerging clades. *Candida auris* clade determination was performed by short tandem repeat (STR) typing assay and was compared to whole-genome sequencing results [21,37]. The selected study strains are detailed in Table 1.

### 2.2. Growth Conditions, Chemical Reagents and Protein Purification

All *C. auris* strains (102 strains) were grown at 30 °C for 48 h, on Sabouraud glucose agar plates (SGA; Oxoid, Thermo Fisher Scientific, Waltham, MA, USA) under aerobic conditions. Optima LC-MS grade Water (H_2_O), Optima LC-MS grade acetonitrile (ACN), Optima LC-MS grade formic acid (FA) were purchased from Fisher Scientific (Landsmeer, The Netherlands). A standard microbial culturing loop was used for sampling, and fungal biomass equivalent to a ‘full loop’ (partially, multiple colonies from the same plate) was collected from culture plates and transferred to a micro vial. Subsequently the cells were dispensed in a pre-incubation solution (Thermo Fisher Scientific, proprietary, Waltham, MA, USA) and following a short centrifugation step the supernatant was discarded and the pellet was suspended to 100 µL of incubation solution (Thermo Fisher Scientific, proprietary). The microbes were incubated for 20 min (vortexed once at 10 min for 2 s), followed by sonication for one minute. Cells were diluted with 100 µL dilution buffer (Thermo Fisher Scientific, proprietary) and centrifuged for 5 min at 12,000× *g* at room temperature. The supernatant was collected in low protein binding (LBE) Eppendorf tube and stored at −20 °C if not immediately used for LC-MS analysis.

### 2.3. RP4H SPE Protocol

The whole cell extract (WCE) was diluted with SPE buffer-1 (Thermo Fisher Scientific, proprietary) before SPE (solid-phase extraction) cleanup. RP4H SPE tips (Thermo Fisher Scientific, proprietary) were placed in a 96-well plate and conditioned and equilibrated with 50 µL proprietary SPE buffer-2 (Thermo Fisher Scientific, proprietary), respectively. Centrifuge (Laboratory centrifuge 4-15C, Thermo Fisher Scientific, Osterode, Germany) capable of handling two 96-well plates was used for centrifugation at 2000× *g* for 2 min at room temperature. Diluted WCE (50 µL) was loaded into the SPE tips and centrifuged. The tips were washed with 50 µL of proprietary SPE washing buffer and placed in Stand-Alone Liquid Chromatography (SALC) autosampler plate for further online elution and mass spectrometric (MS) analysis.

### 2.4. LC-MS Analysis and Raw Data Processing

All experiments were performed by micro flow LC-MS system. The LC system used was stand-alone liquid chromatography (SALC) (Thermo Fisher Scientific). SALC was connected to a Q Exactive™ HF Hybrid Quadrupole-Orbitrap^TM^ mass spectrometer (Thermo Fisher Scientific). The SALC and mass spectrometer were controlled by Xcalibur software version 3.0 (Thermo Fisher Scientific). The mass spectrometer was operated in positive electrospray ionization (+ ESI) mode. The raw data were deconvoluted online to monoisotopic masses i.e., list of proteoforms with a proprietary algorithm. Subsequently, the data were processed with in-house algorithms for building of the database and discrimination of mycobacteria species. Chromatographic separation of proteins was achieved online using SALC through SPE tips and eluted proteins were subjected to MS analysis. Mass analysis of proteins was performed in the *m*/*z* range from 450 to 2000 *m*/*z* for full scan mode. Average mass accuracy was <2 ppm. Pre-processing of the mass spectra was performed by Thermo Fisher Scientific proprietary software to deconvolute raw spectra in *m*/*z* space into monoisotopic protein masses between 5 and 40 kDa. Measured monoisotopic masses from each measurement were mass aligned to construct a list of consensus markers.

### 2.5. Pre-Collected Taxon Database

The pre-collected taxon database contains reference spectra for a wide number of clinical bacterial and yeast species including *C. auris*, 22 strains and one type strain for each taxon of the *C. haemulonis* species complex (*C. haemulonis s. str, C. duobushaemulonis* and *C. pseudohaemulonis*). The MS1 database comprises 6 technical replicates analyzed per strain (covering both biological and technical reproducibility).

### 2.6. Classification and Species Identification

Data analysis workflow employed a classification algorithm (Thermo Fisher Scientific proprietary) matching MS1 spectrum of an unknown sample against diagnostic spectral features (differential proteoforms) in the pre-collected taxon database. The classification algorithms sorted the species by distance of the matched features of the measured spectra and subsequently delivered species level identifications as end-result. Feature selection based on mass frequencies was applied to select top consensus markers that have the most predictive value for the differentiation of the target entities. Mass frequencies were depicted in a heat map to visualize the data and subsequently scored to define classification success. The frequency filtered monoisotopic masses of the study cohort, were further processed in a principal component analysis (PCA), followed by an affinity clustering approach. The resulting outcome was visualized in the observed n-dimensional PCA space relative to the distance of the obtained protein masses. Post-analytical data processing was conducted in R statistical software, as well partially in Python 3.10 employing various open source and proprietary libraries, which mostly concerned data visualization.

## 3. Results

### 3.1. Species Identification and Throughput

**Figure 1:**
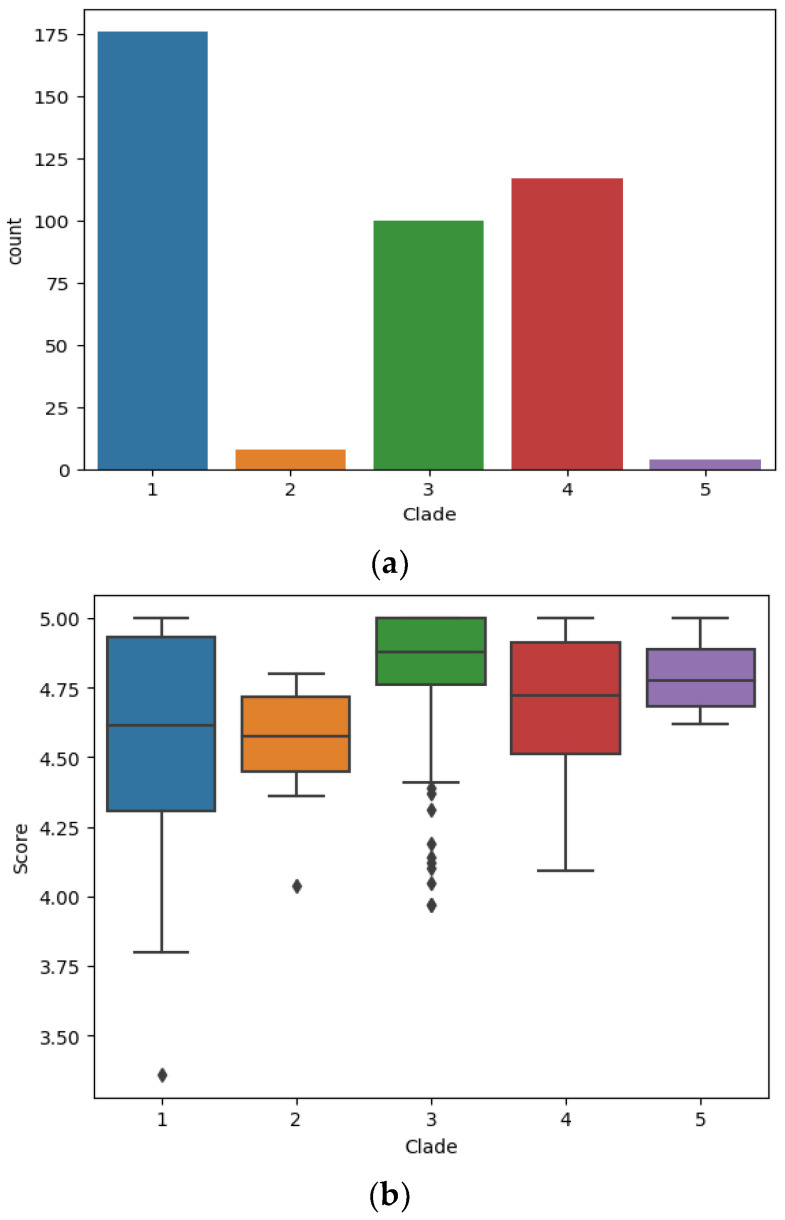
(**a**). Bar charts indicating total count of LC-MS measurements per clade. (**b**). Identification score distribution between five geographic *C. auris* clades. The image depicts a standard boxplot indicating the score distribution for each LC-MS measurement obtained for all strains from each geographic clade respectively. The central bar indicates the median value (Median quantile 1), the box (IQR = Inter quantile range) indicates the range for the majority of scores (vertical expansion), hence the lower and upper section in the box corresponds to the 25% central IQR range respectively (Quantile 2/3). The whiskers (central lines) indicate the range for the outliers (Upper and lower 25% IQR; Quantile 4/5).

All strains of the study panel were subjected to the identification pipeline using all yeast and bacterial reference spectra contained in the pre-collected taxon database. Detailed identification results for each strain within the panel (including identification scores per strain) are presented in Supplementary Table S1 as well a typical example LCMS total ion chromatogram and mass spectrum for different concentrations and elution patters of solvent B (ACN) in Supplementary Figure S1. All *C. auris* strains were correctly identified, none being assigned to related species of the *C. haemulonis* complex, i.e., *C. duobushaemulonis*, *C. haemulonis*, or *C. pseudohaemulonis*. Each isolate from the panel was tested with four replicates in multiple Mass Spectrometry Systems. On average 114 proteoforms were identified over all LC-MS runs (min. 38, max. 155). No discrepancies were noted among the repeated tests, thus demonstrating the precision and reproducibility of the technology. The distribution of the identification scores for individual strains from plate culture within geographic clades is shown in Figure 1. The wide range of score distribution, as depicted in Figure 1, does not impair the final identification, but rather reflects the relative distances of strain-specific proteins that are measured and are compared to the reference database entries. The database contains only those proteins that are specific to the reference strains and accordingly match those shared with the unknown *C. auris* isolate. Therefore, the score result reflects a strain-specific gene expression event at the time of colony sampling (not to be confounded with a transient expression event), and their subsequent proteome profile fit to the database entries. Identification accuracy (%) of strains in each clade and the number of strains identified from plate is provided in Table 2.

### 3.2. Clade and Lineage Identification

*Candida auris* strains representing all major geographic clades of South American, South Asian, African, East Asian and Iranian origin, with most major genotypes (lineages) representing those clades, as identified in a previous study [37], were analyzed. Lineage identification is clearly demonstrated as the potential capability and capacity of the Orbitrap technology.

**Figure 2:**
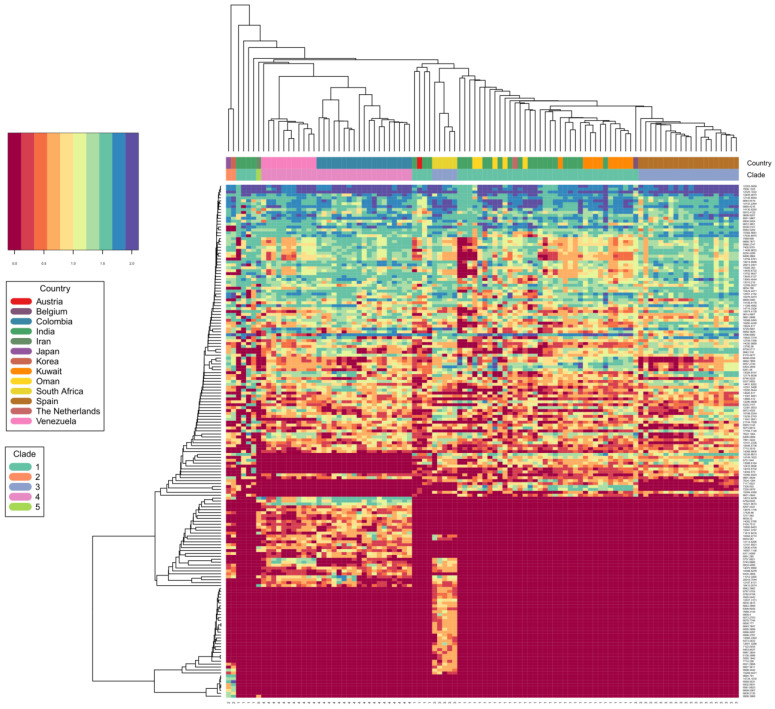
*C. auris* clade differentiation using monoisotopic mass measurements depicted as heat map. Color scale ranges from blue (max signal) to dark red (no signal), representing abundance of measured monoisotopic masses in each strain. Clade specific differential protein masses are visible from the rectangular vertical boxes indicating the geographic affiliation and clade assignment and its vertically associated dendrogram indicating observed protein masses (columns vs. rows). X-axis indicating clade assignment and y-axis indicating observed MS1 protein masses.

**Figure 3:**
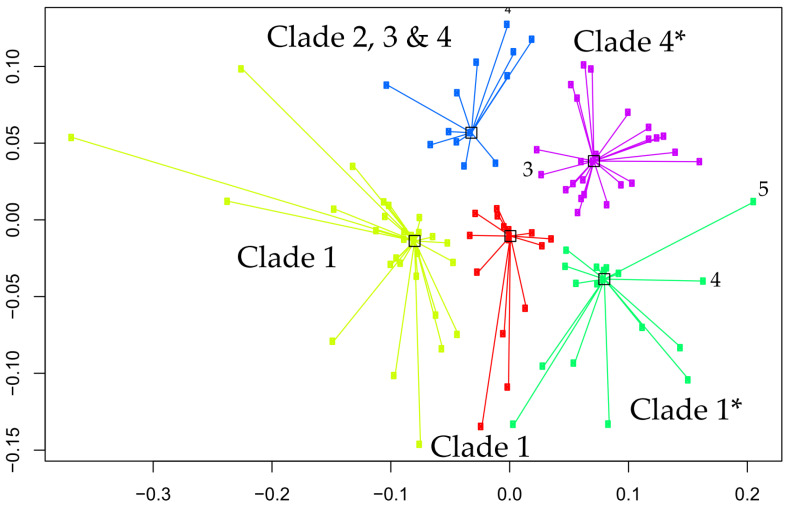
Affinity clustering using combined *C. auris* strain spectra scaled into the n-dimensional PCA space. Numbers corresponding to the identified clusters correspond to the geographic clades, 1 = SAS, 2 = EAS, 3 = SAF, 4 = SAM and 5 = IRN. Axes are dimensionless and relative to within and between cluster distances. Yellow cluster = clade 1, red cluster = clade 1, green cluster = clade 1 with two outlier strains (clade 4,5), purple cluster = clade 4 with one outlier strain (clade 3) and blue cluster = mixed clade assignment (2, 3 and 4). Note, outliers are solely based on affinity clustering, and do not represent the actual classification success (Table 2). Accordingly, this represents higher proportions of shared masses between outliers with the incorrect clade, but do not reversely correlate with specific biomarkers that allow subsequent correct clade prediction with the vendor proprietary classifier. * clusters with outlier isolates.

Mass spectrometric data are here depicted as a heat map, where rows represent individual monoisotopic masses and columns individual strains (technical LC-MS replicates for each strain were merged as a single leave in the horizontal dendrogram) of the study cohort (Figure 2). The protein masses included in the heat map are sorted by their detection frequencies to indicate most differentiating proteoforms. The horizontal colored bar at the top represents the geographic *C. auris* clades (SAS = South East Asia, EAS = East Asia, SAF = South Africa, SAM = South America, IRN = Iranian). The shared differential masses correspond to particular strains. The high performance of the Orbitrap Mass Spectrometer in *C. auris* identification down to the clade; and the individual strain level is outlined by highly specific mass (proteoforms) signatures and matches with geographic clades. The frequency legend ranges from 0 (red) to 2 (blue), indicating if a mass was observed in a sample at a high to low frequency ratio, respectively. The specific proteoforms unique to a clade can be assessed algorithmically but not visually in a precise manner, however the prominent and mostly unique blocks at the bottom of Figure 2 corresponding e.g., to the SAF and SAM clade depict proteoforms consistently recorded for these geographies and samples and which can be termed differential. The high dissimilarities between the clades, as observed by the PCA and followed by subsequent affinity clustering indicate the clade-specific predictive value of the obtained mass spectrometry data, relative to its genomic reference [33,37]. Affinity clustering using combined *C. auris* strain spectra scaled into an n-dimensional PCA space (Figure 3) depicting high dissimilarity between geographic clades 1, 3 and 4, and confirm the high-resolution of Orbitrap Mass Spectral data in differentiating the geographic clades of *C. auris*. While the South Asian clade I, the East Asian clade II and the Iranian clade V agglomerate at a lower level of dissimilarity, which is visualized in their almost identical horizontal position in the plot, the African clade III and the South American clade IV display a wider distance to clades I, II and V, respectively. The results indicate a clear separation of the major geographic entities Asia, Africa and South America.

## 4. Discussion

Correct pathogen identification is of indisputable clinical significance, assisting the clinicians to select appropriate antifungal drugs for the patients and to install timely infection control measures. Mass spectrometry (MALDI-TOF) has entered many clinical microbiology laboratories during the past decade, gradually replacing biochemical methods as the preferred tool for pathogen identification. The technique provides a relatively easy-to-use and fast platform but is yet limited in identification accuracy in many taxonomically challenging cases [35,36].

We applied the advanced Orbitrap^TM^ high-resolution mass spectrometric technology for identification of *C. auris*, using protein analysis at a level extending beyond traditional MALDI-TOF methods. Coupled to liquid chromatography (LC) for initial separation, electrospray ionization (ESI) allows proteins in solution to be transferred into the gas phase for subsequent ionization and introduction into the mass spectrometer (LC-MS). Mass analysis can then be performed for either the intact mass (MS) or fragment ions via tandem mass spectrometry (MS/MS). Orbitrap^TM^ mass analyzer features high resolution (up to 200,000), high mass accuracy (2–5 ppm), a high mass-to-charge ratio of 6000, and a dynamic range greater than 104. The other major advantage of the technology is its high selectivity since it measures the exact mass of a compound allowing even minor changes in structure such as a translated single nucleotide polymorphism into an amino acid change, to be distinguished. Our study shows that the technology enables correct identification of all *C. auris* isolates with a high level of confidence (99.6%). The Orbitrap^TM^’s ability to identify *C. auris* from all clades and to discriminate between *C. auris* and the members the *haemulonis* species complex (*C. haemulonis s.str., C. duobushaemulonis and C. pseudohaemulonis*) was superior to the currently available phenotypical technologies including the latest Vitek 2 (version 8.01) yeast identification system. The latter was able to correctly identify most of the South Asian *C. auris* strains but misidentified East Asian strains and gave a low discrimination result for the South American clade [38,39]. Although the number of isolates in each clade was limited, the Orbitrap^TM^ high-resolution mass spectrometric technology performance appears to be equivalent to the *C. auris* clades obtained from genomic methods, with 99.6% identification accuracy (Table 2) for isolates from all clades (Figure 2 and Figure 3). While our results show a clear separation of the major geographic entities Asia, Africa and South America, our PCA analysis and subsequent affinity clustering indicate that particularly the South Asian (I), East Asian (II) and Iranian (V) clades are proteomically closely related, a result contrasted by genomic data [3] where the African clade (III) with strains from South Africa affiliate to strains from a Spanish outbreak closer to the East Asian clade (II). Similar to the genomic typing schemes [37,40], long branches in the affinity clustering indicate strains that appear as outliers or typical strains relative to those strains centralizing with similar or even distances around a cluster, hence forming a representative central entity. These specific isolates require further attention, regardless of the identification technology if they represent true biological deviants or technical artifacts. However, an example can be given for the isolate from Iran (clade V), which in the genomic typing scheme defines its own clade [21]. This strain affiliates with a very long branch to the ‘green’ sub cluster of the South Asian clade (Figure 3) confirming its uniqueness among the study cohort and particularly among the strains of Asian origin. However, further investigations are required on some isolates with similar distances, e.g., to the ‘yellow’ cluster, in order to understand the exact potential discrepancies and reasons between the genomic vs. proteomic typing results for a very few outliers. This might also be applicable to some of the strains in the well-defined ‘blue’ cluster (African clade III), which associate to some strains originating from the South and East Asian clade (I and II). This is probably explained by the large Indian population and travel between India/Pakistan and South Africa. Accordingly, our proteomic typing results may reflect a high capability of tracking strains of the same origin but isolated in different geographic locations. Precise matching and alignment of typing schemes is required in the future to build on these results. Obtaining congruency to the genomic reference, particularly to either fast typing [37] or next generation sequencing methods employing single nucleotide polymorphism or k-mer [41,42] based classification approaches, not only allows for fast incorporation of novel isolates into a mass spectra database, but also significantly reduces false identifications and classification artifacts of unknown strains associated to such newly described clades or lineages.

While workflow efficiency to identify groups of organisms are not always comparable between unrelated technology platforms, it is important to understand which level of taxonomic or classification resolution can be obtained by which method and how time-to-results are impacted to obtain an equivalent clinical test result. A prime example is the comparison between mass spectrometry and sequencing technologies, where next generation sequencing technologies are now widespread in advanced clinical laboratories and are precisely employed to answer questions so far exclusively reserved to genome sequencing approaches. Even though mass spectrometry and (next generation) sequencing-based workflows are not directly comparable, their final end-results, i.e., the identification of unknown clinical microbes, are well comparable. While MALDI-TOF and high-resolution mass spectrometry workflows can be directly comparable in achieving fast species-level identification, clade (and lineage) identification cannot be achieved by MALDI-TOF. Accordingly, time-to-results must be contrasted to the genomic reference workflow to obtain the equivalent level of taxonomic resolution.

Even though significant advancement in next generation sequencing workflows, library chemistry composition, adaptor design and adaptor combinatorics, sample scalability (e.g., 96-well format) and automation has been achieved over recent years, the standard next generation sequencing workflow remains highly time-consuming as it includes many delicate quality-control steps, particularly for multiplexed sample runs [43]. Even under the most liberal and fastest automated turnaround time workflows, DNA purification, library construction, fragmentation and normalization, multiplexing, cluster generation, total sequencing run-time, subsequent raw data processing (e.g. base-calling and filtering) and subsequently quality controlled raw data pipelining in order to obtain final identification (in the present case *C. auris* geographic clades) cannot be achieved within less than ~24 h [43]. Even the fastest currently known NGS workflows employing e.g., nanopore sequencing, such as ‘read-until’ or ‘real-time’ approaches are not yet scalable and lack efficiency and simplicity in application [44,45,46]. Our methodology in contrast is able to return a result within approximately 60 min. Accordingly, the potential future of the Orbitrap^TM^ high resolution mass spectrometric technology is to provide short time-to-results in obtaining clade or lineage identification, furthermore also by MS/MS fragmentation experiments to actually identify the respective protein via reverse search engines, and to compare an unknown sample to its genomic reference obtained by next generation sequencing.

## 5. Conclusions

The application of the high resolution Orbitrap^TM^ mass spectrometer, for accurate identification of newly emerging species such as *C. auris* with the potential to more in-depth identification down to lineages within the species offers an attractive alternative to currently existing technology platforms.

## Figures and Tables

**Table 1:** Selected *C. auris* strains with country of origin.

Country of Origin	Number of Strains	Genomic Clade
Austria	1	(Clade I) * SAS
Belgium	1	(Clade I) SAS
India	26	(Clade I) SAS
Kuwait	10	(Clade I) SAS
Oman	5	(Clade I) SAS
The Netherlands	1	(Clade I) SAS
Japan	1	(Clade II) * EAS
Korea	1	(Clade II) EAS
Spain	20	(Clade III) SAS
South Africa	5	(Clade III) * SAF
Colombia	19	(Clade IV) * SAM
Venezuela	11	(Clade IV) SAM
Iran	1	(Clade V) * IRN
Total	102	

* SAS: South Asia, EAS: East Asia, SAF: South Africa, SAM: South America, IRN: Iran.

**Table 2:** Identification accuracy of *C. auris* strains.

Clade	ID Accuracy (%)	# of Strains	Standard Error of Mean	Standard Deviation	Mean Score	# of Replicates	# of Strains Identified	Sensitivity (%)
Clade I (SAS)	99.6	44	±0.02	0.35	4.57	176	44	99.6
Clade II (EAS)	100	2	±0.08	0.25	4.53	8	2	100
Clade III (SAF)	100	25	±0.02	0.26	4.79	100	25	100
Clade IV (SAM)	100	30	±0.02	0.25	4.67	120	30	100
Clade V (IRN)	100	1	±0.08	0.16	4.79	4	1	100

## Data Availability

All original data are available from the authors. No large datasets were generated.

## Supplementary Materials

The following supporting information can be downloaded at: https://www.mdpi.com/article/10.3390/jof9020267/s1, Figure S1: Example of liquid chromatography mass spectrometry spectra with gradient elution; Table S1: Detailed identification results for each strain within the panel (including identification scores per strain).

## References

[1] Lee W.G., Shin J.H., Uh Y., Kang M.G., Kim S.H., Park K.H., Jang H.C. (2011). First three reported cases of nosocomial fungemia caused by Candida auris. J. Clin. Microbiol..

[2] Chowdhary A., Sharma C., Duggal S., Agarwal K., Prakash A., Singh P.K., Jain S., Kathuria S., Randhawa H.S., Hagen F. (2013). New clonal strain of Candida auris, Delhi, India. Emerg. Infect. Dis..

[3] Emara M., Ahmad S., Khan Z., Joseph L., Al-Obaid I., Purohit P., Bafna R. (2015). Candida auris candidemia in Kuwait, 2014. Emerg. Infect. Dis..

[4] Chowdhary A., Sharma C., Meis J.F. (2017). Candida auris: A rapidly emerging cause of hospital-acquired multidrug-resistant fungal infections globally. PLoS Pathog..

[5] Saris K., Meis J.F., Voss A. (2018). Candida auris. Curr. Opin. Infect. Dis..

[6] Meis J.F., Chowdhary A. (2018). Candida auris: A global fungal public health threat. Lancet Infect. Dis..

[7] Shastri P., Shankarnarayan S.A., Oberoi J., Rudramurthy S.M., Wattal C., Chakrabarti A. (2020). Candida auris candidaemia in an intensive care unit—Prospective observational study to evaluate epidemiology, risk factors, and outcome. J. Crit. Care.

[8] Hyde K.D., Al-Hatmi A.M.S., Andersen B., Boekhout T., Buzina W., Dawson T.L., Eastwood D.C., Jones E.B.G., de Hoog S., Kang Y. (2018). The world’s ten most feared fungi. Fungal Divers..

[9] https://www.cdc.gov/drugresistance/pdf/threats-report/2019-ar-threats-report-508.pdf.

[10] Ruiz-Gaitan A., Moret A.M., Tasias-Pitarch M., Aleixandre-Lopez A.I., Martinez-Morel H., Calabuig E., Salavert-Lleti M., Ramirez P., Lopez-Hontangas J.L., Hagen F. (2018). An outbreak due to Candida auris with prolonged colonisation and candidaemia in a tertiary care European hospital. Mycoses.

[11] Schelenz S., Hagen F., Rhodes J.L., Abdolrasouli A., Chowdhary A., Hall A., Ryan L., Shackleton J., Trimlett R., Meis J.F. (2016). First hospital outbreak of the globally emerging Candida auris in a European hospital. Antimicrob. Resist. Infect. Control.

[12] Alfouzan W., Ahmad S., Dhar R., Asadzadeh M., Almerdasi N., Abdo N.M., Joseph L., De Groot T., Alali W.Q., Khan Z. (2020). Molecular epidemiology of Candida auris outbreak in a major secondary-care hospital in Kuwait. J. Fungi.

[13] Mohsin J., Weerakoon S., Ahmed S., Puts Y., Al Balushi Z., Meis J.F., Al-Hatmi A.M.S. (2020). A Cluster of Candida auris blood stream infections in a tertiary care hospital in Oman from 2016 to 2019. Antibiotics.

[14] Calvo B., Melo A.S., Perozo-Mena A., Hernandez M., Francisco E.C., Hagen F., Meis J.F., Colombo A.L. (2016). First report of Candida auris in America: Clinical and microbiological aspects of 18 episodes of candidemia. J. Infect..

[15] Morales-Lopez S.E., Parra-Giraldo C.M., Ceballos-Garzon A., Martinez H.P., Rodriguez G.J., Alvarez-Moreno C.A., Rodriguez J.Y. (2017). Invasive infections with multidrug-resistant yeast Candida auris, Colombia. Emerg. Infect. Dis..

[16] Kathuria S., Singh P.K., Sharma C., Prakash A., Masih A., Kumar A., Meis J.F., Chowdhary A. (2015). Multidrug-resistant Candida auris misidentified as Candida haemulonii: Characterization by matrix-assisted laser desorption ionization-time of flight mass spectrometry and DNA sequencing and its antifungal susceptibility profile variability by Vitek 2, CLSI broth microdilution, and Etest method. J. Clin. Microbiol..

[17] Mizusawa M., Miller H., Green R., Lee R., Durante M., Perkins R., Hewitt C., Simner P.J., Carroll K.C., Hayden R.T. (2017). Can multidrug-resistant Candida auris be reliably identified in clinical microbiology laboratories?. J. Clin. Microbiol..

[18] Kenters N., Kiernan M., Chowdhary A., Denning D.W., Pemán J., Saris K., Schelenz S., Tartari E., Widmer A., Meis J.F. (2019). Control of Candida auris in healthcare institutions: Outcome of an International Society for Antimicrobial Chemotherapy expert meeting. Int. J. Antimicrob. Agents.

[19] Caceres D.H., Forsberg K., Welsh R.M., Sexton D.J., Lockhart S.R., Jackson B.R., Chiller T. (2019). Candida auris: A review of recommendations for detection and control in healthcare settings. J. Fungi.

[20] Lockhart S.R., Etienne K.A., Vallabhaneni S., Farooqi J., Chowdhary A., Govender N.P., Colombo A.L., Calvo B., Cuomo C.A., Desjardins C.A. (2017). Simultaneous emergence of multidrug-resistant Candida auris on three continents confirmed by whole-genome sequencing and epidemiological analyses. Clin. Infect. Dis..

[21] Spruijtenburg B., Badali H., Abastabar M., Mirhendi H., Khodavaisy S., Sharifisooraki J., Armaki M.T., de Groot T., Meis J.F. (2022). Confirmation of fifth Candida auris clade by whole genome sequencing. Emerg. Microbes Infect..

[22] Chow N.A., Gade L., Tsay S.V., Forsberg K., Greenko J.A., Southwick K.L., Barrett P.M., Kerins J.L., Lockhart S.R., Chiller T.M. (2018). Multiple introductions and subsequent transmission of multidrug-resistant Candida auris in the USA: A molecular epidemiological survey. Lancet Infect. Dis..

[23] Oladele R., Uwanibe J.N., Olawoye I.B., Ettu A.O., Meis J.F., Happi C.T. (2022). Emergence and genomic characterization of multidrug resistant Candida auris in Nigeria, West Africa. J. Fungi.

[24] Zerrouki H., Ibrahim A., Rebiahi S.A., Elhabiri Y., Benhaddouche D.E., de Groot T., Meis J.F., Rolain J.M., Bittar F. (2022). Emergence of Candida auris in intensive care units in Algeria. Mycoses.

[25] de Almeida J.N., Brandão I.B., Francisco E.C., de Almeida S.L.R., de Oliveira Dias P., Pereira F.M., Ferreira F.S., de Andrade T.S., de Miranda Costa M.M., de Souza Jordão R.T. (2021). Axillary Digital Thermometers uplifted a multidrug-susceptible Candida auris outbreak among COVID-19 patients in Brazil. Mycoses.

[26] Hamprecht A., Barber A.E., Mellinghoff S.C., Thelen P., Walther G., Yu Y., Neurgaonkar P., Dandekar T., Cornely O.A., Martin R. (2019). Candida auris in Germany and previous exposure to foreign healthcare. Emerg. Infect. Dis..

[27] Borman A.M., Johnson E.M. (2020). Candida auris in the UK: Introduction, dissemination, and control. PLoS Pathog..

[28] Chowdhary A., Prakash A., Sharma C., Kordalewska M., Kumar A., Sarma S., Tarai B., Singh A., Upadhyaya G., Upadhyay S. (2018). A multicentre study of antifungal susceptibility patterns among 350 Candida auris isolates (2009-17) in India: Role of ERG11 and FKS1 genes in azole and echinocandin resistance. J. Antimicrob. Chemother..

[29] Bruno M., Kersten S., Bain J.M., Jaeger M., Rosati D., Kruppa M.D., Lowman D.W., Rice P.J., Graves B., Ma Z. (2020). Transcriptional and functional insights into the host immune response against the emerging fungal pathogen Candida auris. Nat. Microbiol..

[30] Yan L., Xia K., Yu Y., Miliakos A., Chaturvedi S., Zhang F., Chen S., Chaturvedi V., Linhardt R.J. (2020). Unique Cell Surface Mannan of Yeast Pathogen Candida auris with Selective Binding to IgG. ACS Infect. Dis..

[31] Kumar M., Singh A., Kumari S., Kumar P., Wasi M., Mondal A.K., Rudramurthy S.M., Chakrabarti A., Gaur N.A., Gow N.A.R. (2020). Sphingolipidomics of drug resistant Candida auris clinical isolates reveal distinct sphingolipid species signatures. Biochim. Biophys. Acta Mol. Cell Biol. Lipids.

[32] Girard V., Mailler S., Chetry M., Vidal C., Durand G., van Belkum A., Colombo A.L., Hagen F., Meis J.F., Chowdhary A. (2016). Identification and typing of the emerging pathogen Candida auris by matrix-assisted laser desorption ionisation time of flight mass spectrometry. Mycoses.

[33] Vatanshenassan M., Boekhout T., Mauder N., Robert V., Maier T., Meis J.F., Berman J., Then E., Kostrzewa M., Hagen F. (2020). Evaluation of Microsatellite Typing, ITS Sequencing, AFLP Fingerprinting, MALDI-TOF MS, and Fourier-Transform Infrared Spectroscopy Analysis of Candida auris. J. Fungi.

[34] Chow N.A., Muñoz J., Gade L., Berkow E.L., Li X., Welsh R.M., Forsberg K., Lockhart S.R., Adam R., Alanio A. (2020). Tracing the Evolutionary History and Global Expansion of Candida auris Using Population Genomic Analyses. mBio.

[35] Buil J.B., van der Lee H.A.L., Curfs-Breuker I., Verweij P.E., Meis J.F. (2019). External Quality Assessment Evaluating the Ability of Dutch Clinical Microbiological Laboratories to Identify Candida auris. J. Fungi.

[36] Dewaele K., Lagro K., Frans J., Hayette M.P., Vernelen K. (2019). Hospital Laboratory Survey for Identification of Candida auris in Belgium. J. Fungi.

[37] De Groot T., Puts Y., Berrio I., Chowdhary A., Meis J.F. (2020). Development of Candida auris short tandem repeat typing and its application to a Global Collection of Isolates. mBio.

[38] Ambaraghassi G., Dufresne P.J., Dufresne S.F., Vallières É., Muñoz J.F., Cuomo C.A., Berkow E.L., Lockhart S.R., Luong M.L. (2019). Identification of Candida auris by use of the updated Vitek 2 Yeast Identification System, Version 8.01: A Multilaboratory Evaluation Study. J. Clin. Microbiol..

[39] Tan Y.E., Teo J.Q., Rahman N.B.A., Ng O.T., Kalisvar M., Tan A.L., Koh T.H., Ong R.T.H. (2019). Candida auris in Singapore: Genomic epidemiology, antifungal drug resistance, and identification using the updated 8.01 VITEK^®^2 system. Int. J. Antimicrob. Agents.

[40] de Groot T., Spruijtenburg B., Parnell L.A., Chow N.A., Meis J.F. (2022). Optimization and validation of Candida auris short tandem repeat analysis. Microbiol. Spectr..

[41] Lepuschitz S., Weinmaier T., Mrazek K., Beisken S., Weinberger J., Posch A.E. (2020). Analytical Performance Validation of Next-Generation Sequencing Based Clinical Microbiology Assays Using a K-mer Analysis Workflow. Front. Microbiol..

[42] Pornputtapong N., Acheampong D.A., Patumcharoenpol P., Jenjaroenpun P., Wongsurawat T., Jun S.R., Yongkiettrakul S., Chokesajjawatee N., Nookaew I. (2020). KITSUNE: A Tool for Identifying Empirically Optimal K-mer Length for Alignment-Free Phylogenomic Analysis. Front. Bioeng. Biotechnol..

[43] Illumina Product Support Webpage: Run Time Estimates on the Illumina Sequencing Platforms. https://support.illumina.com/bulletins/2017/02/run-time-estimates-for-each-sequencing-step-on-illumina-sequenci.html.

[44] Loose M., Malla S., Stout M. (2016). Real-time selective sequencing using nanopore technology. Nat. Methods.

[45] Edwards H.S., Krishnakumar R., Sinha A., Bird S.W., Patel K.D., Bartsch M.S. (2019). Real-Time Selective Sequencing with RUBRIC: Read Until with Basecall and Reference-Informed Criteria. Sci. Rep..

[46] Naccache S.N., Federman S., Veeraraghavan N., Zaharia M., Lee D., Samayoa E., Bouquet J., Greninger A.L., Luk K.C., Enge B. (2014). A cloud-compatible bioinformatics pipeline for ultrarapid pathogen identification from next-generation sequencing of clinical samples. Genome Res..

