# Supplementary material for: Fast and Accurate Identification of Candida auris by High Resolution Mass Spectrometry
Source: J Fungi (Basel). 2023 Feb 16;9(2):267. doi: 10.3390/jof9020267 (PMC9966097; doi:10.3390/jof9020267)
Supplement: Supplementary file 1 [file jof-09-00267-s001.zip › jof-2169414-supplementary.pdf]

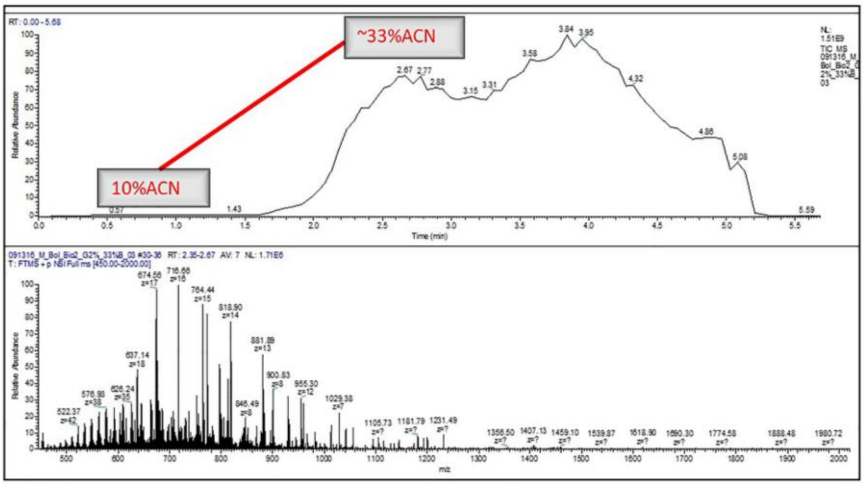

Figure S1A

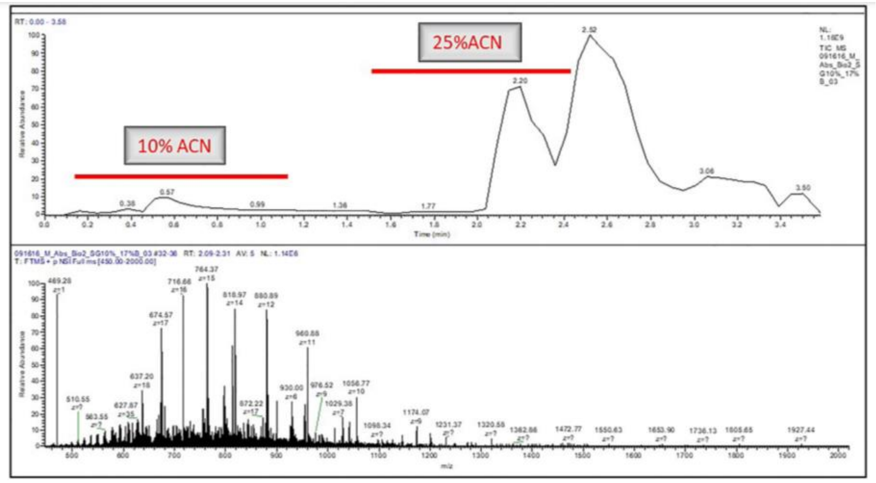

Figure S1B

Supplemental Figure S1 – Example LCMS mass spectra with gradient elution (A) 4 μL/min 2% solvent B (ACN) > to 33% B in 5 min and isocratic elution (B) with 2% solvent B (ACN) for 2 min > stepwise ramp to 25 % B in 3 min. Top graph TIC, bottom graph mass spectrum for Figure S1A and S1B respectively. LCMS: liquid chromatography mass spectrometry, ACN: acetonitrile, TIC: total ion chromatogram.

Supplemental Table S1- Identification results obtained from classifying *C. auris* strains mass spectra. Score refers to the classification success obtained with the classifier to predict the species label (range 0 (min.) to 5 (max.)).

*Species identifier (Strain collection)	Clade	Country	Correct ID	Average score ± std. error of mean
**53764**	Clade I (**SAS)	Austria	*C.auris*	4.7± 0.21
**53156**	Clade I (SAS)	Belgium	*C.auris*	4.6± 0.21
**53124**	Clade I (SAS)	India	*C.auris*	4.4± 0.27
**53099**	Clade I (SAS)	India	*C.auris*	4.4± 0.17
**53106**	Clade I (SAS)	India	*C.auris*	5.0± 0.05
**53103**	Clade I (SAS)	India	*C.auris*	4.6± 0.17
**53120**	Clade I (SAS)	India	*C.auris*	4.1± 0.12
**53100**	Clade I (SAS)	India	*C.auris*	4.0± 0.12
**53122**	Clade I (SAS)	India	*C.auris*	4.6± 0.23
**53127**	Clade I (SAS)	India	*C.auris*	4.9± 0.1
**53104**	Clade I (SAS)	India	*C.auris*	4.7± 0.21
**53126**	Clade I (SAS)	India	*C.auris*	4.9± 0.19
**53119**	Clade I (SAS)	India	*C.auris*	4.7± 0.19
**53123**	Clade I (SAS)	India	*C.auris*	4.4± 0.07
**53744**	Clade I (SAS)	India	*C.auris*	4.7± 0.23
**53748**	Clade I (SAS)	India	*C.auris*	4.5± 0.0
**53749**	Clade I (SAS)	India	*C.auris*	4.8± 0.06
**53750**	Clade I (SAS)	India	*C.auris*	4.9± 0.07
**53751**	Clade I (SAS)	India	*C.auris*	4.5± 0.28
**53752**	Clade I (SAS)	India	*C.auris*	4.8± 0.43
**53753**	Clade I (SAS)	India	*C.auris*	4.6± 0.17
**53754**	Clade I (SAS)	India	*C.auris*	4.5± 0.07
**53767**	Clade I (SAS)	India	*C.auris*	4.8± 0.00
**53769**	Clade I (SAS)	India	*C.auris*	4.7± 0.29
**53775**	Clade I (SAS)	India	*C.auris*	4.6± 0.10
**53776**	Clade I (SAS)	India	*C.auris*	4.7± 0.09
**53793**	Clade I (SAS)	India	*C.auris*	4.7± 0.28
**53794**	Clade I (SAS)	India	*C.auris*	4.4± 0.00
**53178**	Clade I (SAS)	Kuwait	*C.auris*	5.1± 0.21

**53183**	Clade I (SAS)	Kuwait	*C.auris*	5.0± 0.25
**53184**	Clade I (SAS)	Kuwait	*C.auris*	4.9± 0.26
**53186**	Clade I (SAS)	Kuwait	*C.auris*	4.6± 0.25
**53181**	Clade I (SAS)	Kuwait	*C.auris*	4.6± 0.21
**53179**	Clade I (SAS)	Kuwait	*C.auris*	4.5± 0.32
**53182**	Clade I (SAS)	Kuwait	*C.auris*	4.7± 0.32
**53187**	Clade I (SAS)	Kuwait	*C.auris*	4.8± 0.11
**53180**	Clade I (SAS)	Kuwait	*C.auris*	4.7± 0.08
**53185**	Clade I (SAS)	Kuwait	*C.auris*	4.9± 0.13
**52889**	Clade I (SAS)	Oman	*C.auris*	4.7± 0.20
**52903**	Clade I (SAS)	Oman	*C.auris*	4.9± 0.29
**52904**	Clade I (SAS)	Oman	*C.auris*	4.6± 0.28
**52905**	Clade I (SAS)	Oman	*C.auris*	4.8± 0.15
**53762**	Clade I (SAS)	Oman	*C.auris*	4.8± 0.18
**53102**	Clade III (SAS)	Spain	*C.auris*	4.9± 0.07
**53112**	Clade III (SAS)	Spain	*C.auris*	5.0± 0.08
**53117**	Clade III (SAS)	Spain	*C.auris*	5.0± 0.06
**53095**	Clade III (SAS)	Spain	*C.auris*	5.0± 0.02
**53114**	Clade III (SAS)	Spain	*C.auris*	4.9± 0.22
**53116**	Clade III (SAS)	Spain	*C.auris*	4.5± 0.26
**53130**	Clade III (SAS)	Spain	*C.auris*	5.0± 0.05
**53131**	Clade III (SAS)	Spain	*C.auris*	4.7± 0.22
**53132**	Clade III (SAS)	Spain	*C.auris*	4.7± 0.25
**53094**	Clade III (SAS)	Spain	*C.auris*	4.9± 0.1
**53732**	Clade III (SAS)	Spain	*C.auris*	4.8± 0.19
**53733**	Clade III (SAS)	Spain	*C.auris*	4.9± 0.04
**53734**	Clade III (SAS)	Spain	*C.auris*	4.8± 0.1
**53735**	Clade III (SAS)	Spain	*C.auris*	4.9± 0.03
**53736**	Clade III (SAS)	Spain	*C.auris*	4.9± 0.06
**53737**	Clade III (SAS)	Spain	*C.auris*	4.9± 0.07
**53738**	Clade III (SAS)	Spain	*C.auris*	4.8± 0.02
**53739**	Clade III (SAS)	Spain	*C.auris*	5.0± 0.03
**53740**	Clade III (SAS)	Spain	*C.auris*	4.5± 0.18
**53755**	Clade III (SAS)	Spain	*C.auris*	4.7± 0.26

**53765**	Clade I (SAS)	The Netherlands	*C.auris*	4.7± 0.12
**51006**	Clade II (**EAS)	Japan	*C.auris*	4.4 ± 0.13
**51014**	Clade II (EAS)	Korea	*C.auris*	4.7± 0.10
**53134**	Clade III (**SAF)	South Africa	*C.auris*	4.7± 0.11
**53137**	Clade III (SAF)	South Africa	*C.auris*	4.8± 0.07
**53136**	Clade III (SAF)	South Africa	*C.auris*	4.8± 0.06
**53138**	Clade III (SAF)	South Africa	*C.auris*	4.7± 0.13
**53135**	Clade III (SAF)	South Africa	*C.auris*	4.7± 0.14
**53014**	Clade IV (**SAM)	Colombia	*C.auris*	4.6± 0.14
**53026**	Clade IV (SAM)	Colombia	*C.auris*	4.6± 0.15
**53043**	Clade IV (SAM)	Colombia	*C.auris*	4.8± 0.07
**53021**	Clade IV (SAM)	Colombia	*C.auris*	4.6± 0.21
**53022**	Clade IV (SAM)	Colombia	*C.auris*	4.8± 0.11
**53031**	Clade IV (SAM)	Colombia	*C.auris*	4.7± 0.14
**53032**	Clade IV (SAM)	Colombia	*C.auris*	4.7± 0.13
**53033**	Clade IV (SAM)	Colombia	*C.auris*	4.8± 0.18
**53010**	Clade IV (SAM)	Colombia	*C.auris*	4.7± 0.07
**53027**	Clade IV (SAM)	Colombia	*C.auris*	4.8± 0.16
**53041**	Clade IV (SAM)	Colombia	*C.auris*	4.6± 0.19
**53058**	Clade IV (SAM)	Colombia	*C.auris*	4.7± 0.05
**53016**	Clade IV (SAM)	Colombia	*C.auris*	4.8± 0.06
**53048**	Clade IV (SAM)	Colombia	*C.auris*	4.8± 0.16
**53051**	Clade IV (SAM)	Colombia	*C.auris*	4.7± 0.18
**53160**	Clade IV (SAM)	Colombia	*C.auris*	4.6± 0.05
**53759**	Clade IV (SAM)	Colombia	*C.auris*	4.7± 0.09
**53760**	Clade IV (SAM)	Colombia	*C.auris*	4.8± 0.10
**53761**	Clade IV (SAM)	Colombia	*C.auris*	4.8± 0.09
**53151**	Clade IV (SAM)	Venezuela	*C.auris*	4.6± 0.19
**53143**	Clade IV (SAM)	Venezuela	*C.auris*	4.5± 0.20
**53145**	Clade IV (SAM)	Venezuela	*C.auris*	4.6± 0.19
**53148**	Clade IV (SAM)	Venezuela	*C.auris*	4.6± 0.17
**53153**	Clade IV (SAM)	Venezuela	*C.auris*	4.7± 0.15
**53140**	Clade IV (SAM)	Venezuela	*C.auris*	4.7± 0.16
**53141**	Clade IV (SAM)	Venezuela	*C.auris*	4.7± 0.20

**53147**	Clade IV (SAM)	Venezuela	*C.auris*	4.8± 0.12
**53155**	Clade IV (SAM)	Venezuela	*C.auris*	4.5± 0.13
**53139**	Clade IV (SAM)	Venezuela	*C.auris*	4.6± 0.14
**53142**	Clade IV (SAM)	Venezuela	*C.auris*	4.6± 0.11
**53763**	Clade V (**IRN)	Iran	*C.auris*	4.8± 0.10

*Each sample identifier number refers to a patient sample and there is no repeated entry.

****SAS**: South Asia, **EAS**: East Asia, **SAF**: South Africa, **SAM**: South America, **IRN**: Iran.
